# The Self-Assembly Phenomenon of Poloxamers and Its Effect on the Dissolution of a Poorly Soluble Drug from Solid Dispersions Obtained by Solvent Methods

**DOI:** 10.3390/pharmaceutics11030130

**Published:** 2019-03-19

**Authors:** Joanna Szafraniec, Agata Antosik, Justyna Knapik-Kowalczuk, Krzysztof Chmiel, Mateusz Kurek, Karolina Gawlak, Joanna Odrobińska, Marian Paluch, Renata Jachowicz

**Affiliations:** 1 Department of Pharmaceutical Technology and Biopharmaceutics, Faculty of Pharmacy, Jagiellonian University Medical College, Medyczna 9, 30-688 Krakow, Poland; Agata.Antosik@uj.edu.pl (A.A.); Mateusz.Kurek@uj.edu.pl (M.K.); Mfjachow@cyf-kr.edu.pl (R.J.); 2 Department of Physical Chemistry and Electrochemistry, Faculty of Chemistry, Jagiellonian University, Gronostajowa 2, 30-387 Krakow, Poland; Gawlak@chemia.uj.edu.pl (K.G.); Odrobinska@chemia.uj.edu.pl (J.O.); 3 Division of Biophysics and Molecular Physics, Institute of Physics, University of Silesia, Uniwersytecka 4, 40-007 Katowice, Poland; Justyna.Knapik-Kowalczuk@smcebi.edu.pl (J.K.-K.); Krzysztof.Chmiel@smcebi.edu.pl (K.C.); Marian.Paluch@us.edu.pl (M.P.); 4 Silesian Center for Education and Interdisciplinary Research, 75 Pulku Piechoty 1a, 41-500 Chorzow, Poland

**Keywords:** bicaludamide, poloxamer, evaporation, spray drying, dissolution enhancement, nanoaggregates, self-assembly

## Abstract

The self-assembly phenomenon of amphiphiles has attracted particular attention in recent years due to its wide range of applications. The formation of nanoassemblies able to solubilize sparingly water-soluble drugs was found to be a strategy to solve the problem of poor solubility of active pharmaceutical ingredients. Binary and ternary solid dispersions containing Biopharmaceutics Classification System (BCS) class II drug bicalutamide and either Poloxamer^®^188 or Poloxamer^®^407 as the surface active agents were obtained by either spray drying or solvent evaporation under reduced pressure. Both processes led to morphological changes and a reduction of particle size, as confirmed by scanning electron microscopy and laser diffraction measurements. The increase in powder wettability was confirmed by means of contact angle measurements. The effect of an alteration of the crystal structure was followed by powder X-ray diffractometry while thermal properties were determined using differential scanning calorimetry. Interestingly, bicalutamide exhibited a polymorph transition after spray drying with the poloxamer and polyvinylpyrrolidone (PVP), while the poloxamer underwent partial amorphization. Moreover, due to the surface activity of the carrier, the solid dispersions formed nanoaggregates in water, as confirmed using dynamic light scattering measurements. The aggregates measuring 200–300 nm in diameter were able to solubilize bicalutamide inside the hydrophobic inner parts. The self-assembly of binary systems was found to improve the amount of dissolved bicalutamide by 4- to 8-fold in comparison to untreated drug. The improvement in drug dissolution was correlated with the solubilization of poorly soluble molecules by macromolecules, as assessed using emission spectroscopy.

## 1. Introduction

The issue of poor solubility of active pharmaceutical ingredients (APIs) is one of the biggest limitations for drug development. It is a matter of concern, as the bioavailability depends on the dissolution of drug in the gastrointestinal fluids. The main determinants of the dissolution kinetics *in vivo* are solubility and surface area of the particles. The solubility is a function of the crystal lattice energy and the affinity of solid phase to the solvent. Thus, three groups of strategies that have been implemented to improve the rate of dissolution and solubility rely on: (1) the reduction of the intermolecular forces in solid phase, (2) the enhancement of the solid–solvent interaction, and (3) the increase of the surface area available for solvation (according to the Noyes–Whitney equation) [1].

Due to the fact that almost 50% of currently marketed drugs and over 70% of new chemical entities exhibit low solubility in water, numerous techniques have been developed to overcome this problem [2]. Common strategies include pH adjustment, formation of salts, cosolvency, formation of cocrystals and inclusion complexes, particle size reduction, supercritical fluid technology (SCF), and self-emulsification [3,4]. Recently, nanotechnology has emerged as a technique that leads to the formation of robust delivery systems. Numerous attempts have been applied to obtain several types of delivery systems, i.e., micelles [5], liposomes [6], capsules [7,8], protein nanocontainers [9], and silica-based nanoparticles [10,11]. Poorly water-soluble drugs have been frequently processed with hydrophilic polymers, as the molecular dispersion of drug molecules within the matrix provides better dissolution of the drug. Moreover, when the systems were further formulated into the nanoparticles, the results were more pronounced [12,13,14].

The main factors affecting the choice of a particular method are the physicochemical characteristics of drugs and carriers. Solid dispersions are commonly formed to enhance the water solubility of APIs; however, the number of marketed products arising from that strategy is rather low. This is a result of the thermal instability of drug and carrier during preparation of systems, a poor *in vitro–in vivo* correlation, and instability during storage [15]. However, the simplicity of preparation, low cost, and great improvements in the dissolution of poorly water-soluble drugs have made the solid dispersions widely investigated. Experimental and theoretical approaches have been involved to determine the thermodynamic properties of APIs dispersed in polymer matrices as well as the mechanisms and factors affecting their stability [16,17,18].

The concept of solid dispersion—one of the earliest methods of solubility enhancement—was introduced in 1961 by Sekiguchi and Obi, who prepared eutectic mixtures containing microcrystalline drug and a water-soluble carrier [19,20,21,22]. Although crystalline forms provide high stability and chemical purity, the lattice energy barrier is the major limitation affecting the dissolution rate. Thus, amorphous carriers such as polyvinylpyrrolidone (PVP) [23,24] and hydroxypropylmethyl cellulose (HPMC) [25,26] have been introduced to prepare amorphous solid dispersions (ASDs). The highly water-soluble amorphous carriers provide stabilization of APIs, increasing the wettability and dispersibility of the drug [27,28,29]. They limit the precipitation of a drug in water; however, the supersaturation may lead to precipitation and recrystallization of APIs, which negatively affects the bioavailability of the drug. To face this problem, surface active agents or self-emulsifiers such as poloxamers (PLXs) [30,31], Tween 80 [32], or sodium lauryl sulfate (SLS) [33] have been introduced. They improve the dissolution rate as well as physical and chemical stability of the supersaturated system. Surfactants or emulsifiers enhance the miscibility and thus limit the recrystallization rate of the drug. Moreover, they are able to absorb onto the outer layer of drug particles or form micelles encapsulating drug particles, effectively preventing drug precipitation [34]. On the other hand, many surfactants can absorb moisture, which may result in phase separation during storage, an increase in drug mobility, and conversion from the amorphous or metastable form to the more stable crystalline one. They may change the physical properties of the matrix, increase the water content and cause adverse side effects *in vivo*. [35] Thus, their use has to be cautious and their amounts well adjusted.

Among the strategies that allow for obtaining solid dispersions, solvent methods are often used. In these techniques the drug and the carrier are dissolved in a volatile solvent such as ethanol [36] or methylene chloride–ethanol mixture [37] that is further evaporated. It requires sufficient solubility of the drug as well as the carrier in the solvent. Moreover, the type of used solvent, the temperature, and rate of its evaporation are of key importance due to the fact that the concentration of residual solvent needs to be below the detection limit after drying. One of the strategies utilized to fulfill that requirement is the use of low-toxicity solvent mixtures, e.g., water with ethanol, which decreases the amount of each solvent in dry formulation. However, this strategy sometimes fails due to insufficient dissolution of components at a given ratio [35]. Usually, a second drying step is applied to completely removed the solvent as it may lower the glass transition temperature, enhancing the recrystallization tendency.

The common feature of evaporation approaches is the removal of small droplets or thin layers of the solvent from different surfaces. It may lead to the crystal growth of oriented morphology as described for droplet evaporative crystallization or microwave-accelerated evaporative crystallization [38,39,40]. The crystallization of the celocoxib–PVP mixture was found to generate drug crystals of improved dissolution characteristics [41]. Other approaches such as the evaporative antisolvent method and supercritical carbon dioxide evaporation were applied to the formation of nanoparticles, drug-loaded micelles, and liposomes characterized by improved dissolution of the drug [42,43].

Commonly used solvent methods include vacuum drying using rotary evaporators [44], spray drying [45], or freeze-drying [46], among others. In rotary evaporators, solvents are removed under reduced pressure, limiting thermal decomposition of the components of the mixture as organic solvent evaporation occurs at low temperature. Spray drying combines four processes, i.e., (1) atomization of the liquid containing dissolved or suspended drug, which is transported into the nozzle and then sprayed onto fine droplets, (2) mixing the liquid with the drying gas, (3) evaporation, and finally (4) separation of obtained particles from the gas using cyclone [47]. Generally, the spray drying process can be applied for the generation of amorphous materials as well as a technique for particle engineering, i.e., particle size reduction [48].

In the work reported herein, we study the self-assembly phenomenon of solid dispersions containing either Poloxamer^®^188 or Poloxamer^®^407 and its effect on dissolution enhancement of the poorly water soluble drug bicalutamide (BCL). Poloxamers are the nonionic surfactants widely used in pharmaceutical formulations as emulsifiers, wetting agents and solubilizers. They have been introduced into solid dispersions to enhance solubility and dissolution profiles of poorly water-soluble APIs from solid dosage forms [49,50]. Bicalutamide was used as a model drug. It is a non-steroidal antiandrogenic drug assigned to Biopharmaceutics Classification System (BCS) class II because of poor water solubility (below 3.7 mg/L) and high membrane permeability (logP = 2.92) [51,52,53]. It is known to exhibit polymorphism and undergo mechanical activation upon milling [54,55,56,57,58]. Obtained results indicate that the formation of solid dispersions by means of solvent methods led to the changes of particles in solid state, i.e., morphological features, increased wettability, phase transition (in case of ternary solid dispersions containing PVP) and partial disruption of crystal lattice. Moreover, the formation of nanoaggregates in aqueous media led to the 4- to 8-fold increase in the amount of dissolved bicalutamide. Emission spectroscopy allowed for a correlation of the effect of dissolution changes with the solubilization related to the variations of molecular structure of used poloxamers.

## 2. Materials and Methods

### 2.1. Materials

Bicalutamide (BCL, *N*-[4-cyano-3-(trifluoromethyl)phenyl]-3-[(4-fluorophenyl)sulfonyl]-2-hydroxy-2-methylpropanamide, 99.8%, Hangzhou Hyper Chemicals Limited, Zhejiang, China) was used as a model drug. Poloxamer^®^188, Poloxamer^®^407 (BASF, Ludwigshafen am Rhein, Germany), and polyvinylpyrrolidone K29/32 (PVP, Ashland, Covington, KY, USA) were used as excipients. Sodium lauryl sulfate (SLS, BASF, Ludwigshafen am Rhein, Germany) was used to prepare dissolution medium. Ethanol (absolute, 99.8%, pure p.a., Avantor Performance Materials, Gliwice, Poland) and methanol (p.a., Chempur, Piekary Slaskie, Poland) were used as solvents. Cyclohexane (ACS, pure p.a., Avantor Performance Materials, Gliwice, Poland) was used as a dispersant in laser diffraction measurements. Perylene (Pe, p.a., Koch-Light Laboratories Ltd., Colnbrook, UK) and pyrene (98%, Sigma-Aldrich, Darmstadt, Germany) were used in fluorescence emission measurements. All chemicals were used as received. Distilled water was used to prepare all of aqueous solutions.

### 2.2. Methods of Preparation of Solid Dispersions

#### 2.2.1. Solvent Evaporation (E)

Bicalutamide (2 g) was mixed with either Poloxamer^®^188 or Poloxamer^®^407 in a 1:1 and 2:1 wt. ratio, placed in the round-bottomed flask, and dissolved in 200 mL of absolute ethanol. The solution was heated up to 40 °C in the water bath and after complete dissolution of the mixture the solvent was evaporated using a Hei-VAP Value rotavapor (Heidolph, Schwabach, Germany). The rotational speed was equal to 200 rpm and the pressure was reduced stepwise to ca. 40 mbar. The dry solid dispersion was transferred to a container and dried under vacuum prior to further characterization. The systems were further labeled as BCL-PLX188 1:1 (E), BCL-PLX188 2:1 (E), BCL-PLX407 1:1 (E) and BCL-PLX407 2:1 (E), respectively.

#### 2.2.2. Spray Drying (SD)

An ethanolic solution containing bicalutamide mixed with the appropriate carrier or carrier mixture (1:1 and 2:1 wt. ratio, respectively) was spray-dried using a Mini Spray Dryer B-191 (Büchi, Flawil, Switzerland). The process was conducted using following parameters: T_inlet_ = 50–53 °C, T_outlet_ = 39–42 °C, aspirator flow 100%, gas flow rate 600 L/min, liquid flow rate 3.4 mL/min, and a 0.7-mm diameter nozzle. The process was carried out under a constant control and the concentration of ethanol was 10-times lower than the flammability limit. The samples were further dried under vacuum to remove residual solvent. The systems were labeled as BCL-PLX188 1:1 (SD), BCL-PLX188 2:1 (SD), BCL-PLX407 1:1 (SD) and BCL-PLX407 2:1 (SD), BCL-PLX188-PVP 2:1:1 (SD), BCL-PLX188-PVP 4:1:1 (SD), BCL-PLX407-PVP 2:1:1 (SD), and BCL-PLX407-PVP 4:1:1 (SD), respectively.

#### 2.2.3. Scanning Electron Microscopy (SEM)

A Phenom Pro desktop electron microscope (PhenomWorld, Thermo Fisher Scientific, Waltham, MA, USA) equipped with a CeB_6_ electron source and backscattered electron detector was used to determine the morphological features of the samples. The acceleration voltage was equal to 10 kV and the magnification was 750× for evaporated samples, 5000× for spray-dried ternary solid dispersions, and 10,000× for zoomed sections. The powder was placed on the conductive adhesive tape previously glued to the specimen mount. The holder for non-conductive samples was used. The excess of sample (loosely bound to the tape) was removed using a stream of argon. The samples were not sputtered prior to the measurement.

#### 2.2.4. Differential Scanning Calorimetry (DSC)

A DSC 1 STARe System (Mettler–Toledo, Greifensee, Switzerland) was used in order to examine the thermal properties of the samples. The measuring device was equipped with a HSS8 ceramic sensor with 120 thermocouples and a liquid nitrogen cooling station. The apparatus was calibrated for temperature and enthalpy using zinc and indium standards. Melting points were determined as the onset of the peak, with the glass transition temperatures as the midpoint of the heat capacity increment. The samples were measured in an aluminum crucible (40 μL). All measurements were carried out with a heating rate equal to 10 K/min.

#### 2.2.5. Powder X-ray Diffraction (PXRD)

The diffraction patterns of the samples were registered using an X-ray diffractometer Mini Flex II (Rigaku, Tokyo, Japan). The angular range 3–70° 2θ was scanned with a scan speed of 5°/min and a step size equal to 0.02. The measurements were carried out using monochromatic Cu Kα radiation (λ = 1.5418 Å) at ambient temperature. The samples in form of powder were placed in a standard glass sample holder without milling prior the measurement.

#### 2.2.6. Laser Diffraction Measurements

A Mastersizer 3000 equipped with a HydroEV unit (Malvern Instruments, Malvern, UK) was used to determine the particle size distribution. The samples were analyzed by the wet method using cyclohexane (reflective index, RI = 1.426) as a dispersant. The cyclohexane was filtered through the G5 sintered disc filter funnel and placed in the beaker. The rotational speed of the mixer was 1500 rpm. The sample in powder form was added until the obscuration reached the given value (between 5% and 20%) and then the measurement was carried out. A Fraunhofer diffraction theory was applied to find the relationship between particle size and light intensity distribution pattern. Reported data represent the averages from 10 series of measurements for each sample.

#### 2.2.7. Fourier Transform Infrared Spectroscopy (FTIR)

A Nicolet iS10 FT-IR spectrometer (Thermo Fisher Scientific, Waltham, MA, USA) equipped with a Smart iTR™ ATR (Attenuated Total Reflectance) sampling accessory with diamond as an ATR crystal was used to collect the vibrational spectra of powders. Spectra were collected within the range 600–4000 cm^−1^ with 4 cm^−1^ resolution. Presented data represent average from 128 scans for each sample.

#### 2.2.8. Dynamic Light Scattering Measurements (DLS)

The size distribution of aggregates formed by the solid dispersions obtained by either evaporation or spray drying was determined using a Zetasizer Nano ZS instrument (Malvern Instruments, Malvern, UK) working at a 173° detection angle. The distribution analysis was performed at 25 °C using the general purpose mode. The powder was weighted, dissolved in water (c_PLX_ = 2.5 mg/mL) and shaken using a KS 130 Basic orbital shaker (IKA, Staufen im Breisgau, Germany) for 24 h. After that the sample was filtered through the 0.45-µm syringe filter and measured without further dilution. The reported data represent the averages of three series of measurements (10–100 runs each) of hydrodynamic diameter and their standard deviations.

#### 2.2.9. Emission Spectroscopy

A SLM 8100 spectrofluorometer of L-geometry (Aminco, Silver Spring, MD, USA) equipped with a 450W xenon lamp as a light source was used to capture the emission spectra. The microliter quantities of the molecular probes (c ≈ 10^−4^ M), i.e., methanolic perylene solution or ethanolic pyrene solution were slowly injected into a milliliter volume of aqueous PLX188 or PLX407 solutions (c_PLX_ = 5 mg/mL) as well to the solid dispersions solutions previously filtered through a 0.45-μm syringe membrane filter and vigorously stirred. The residues of organic solvents were removed by purging the solution with nitrogen. The samples were equilibrated in the dark for at least 12 h and diluted 10 times before the measurement.

#### 2.2.10. Contact Angle Determination

The wettability of binary systems was assessed by the contact angle measurements performed using a DSA255 drop shape analyzer (Krüss, Hamburg, Germany). The sessile drop technique was used. The droplet of distilled water of volume equal to 2 µL was deposited on the surface of powders compressed using an Atlas^TM^ manual 15Ton hydraulic press (Specac, Kent, UK) with a load pressure of 1.5 tons that was applied for each sample for 15 s.

#### 2.2.11. Dissolution Study

Dissolution of BCL was carried out according to the method recommended by the FDA for BCL tablets (1000 mL of 1% SLS, 37 ± 0.5 °C, 50 rpm) in the pharmacopeial paddle dissolution apparatus Vision Elite 8 (Hanson Research, Chatsworth, CA, USA) equipped with a VisionG2 AutoPlus Autosampler. The sink conditions were maintained. Pure drug and binary systems (solid dispersions, physical mixtures), equivalent of 50 mg of BCL were placed into the beakers. The samples were analyzed spectrophotometrically at 272 nm using a UV-1800 spectrofotometer (Shimatzu, Kioto, Japan) equipped with the flow-through cuvettes. The tests were carried out in triplicate and presented results represents averages with their standard deviations.

## 3. Results and Discussion

### 3.1. Solid State Characterization

#### 3.1.1. Size Distribution and Morphology of Particles of Solid Dispersions

**Figure 1:**
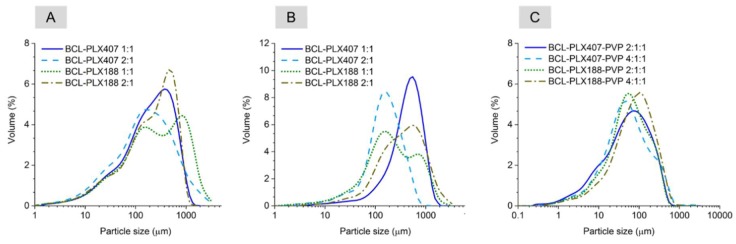
Particle size distribution of solid dispersions obtained by evaporation technique (**A**) and spray drying of binary (**B**) and ternary systems (**C**). BCL: bicalutamide; PLX: poloxamer; PVP: polyvinylpyrrolidone.

The effect of applied processes on the particle size and morphology was studied using both scanning electron microscopy and laser diffraction measurements. The analysis of size distribution of BCL-PLX solid dispersions obtained via evaporation in rotavapor confirmed the heterogeneity of particle size (Figure 1A). Long tails of the distribution curves in the region of small particles were well pronounced. Moreover, the shape of the distribution suggests that several fractions of particles of different sizes were present in the sample, as more than one maximum can be noticed. This is particularly noticeable for the BCL-PLX188 1:1 (E) system, which exhibits a bimodal long-tailed distribution of particle size. This is reflected by great differences in Dx(90) values between the samples, i.e., the point in the size distribution, up to which 90% of the total volume of material in the sample is included. The value was 1190.0 µm for BCL-PLX188 1:1 (E) solid dispersion, while it varied between 630–730 µm for the other evaporated systems. The Dx(10) and Dx(50) values representing the diameter of particles where 10% and a half of the particle population lie below, respectively, did not vary between the corresponding samples; however, the values are greater for BCL-PLX188 1:1 (E) and BCL-PLX407 2:1 (E), which also exhibit long tails in the region of particles that exceeded 1000 µm in length.

**Figure 2:**
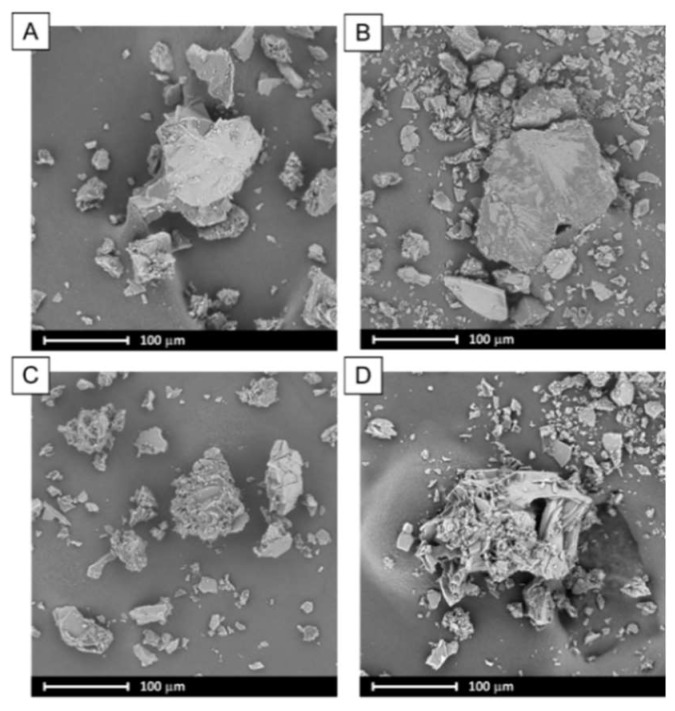
SEM images of binary systems containing bicalutamide and either PLX188 in 1:1 (**A**) and 2:1 (**B**) wt. ratio or PLX407 in 1:1 (**C**) and 2:1 (**D**) wt. ratio obtained using evaporation method.

**Figure 3:**
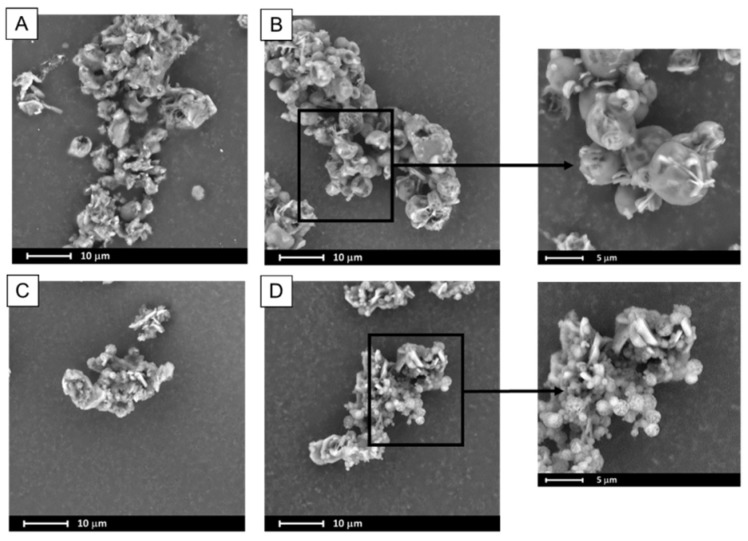
SEM images of ternary systems containing bicalutamide, PVP and either PLX188 in 4:1:1 (**A**) and 2:1:1 (**B**) wt. ratio or PLX407 in 4:1:1 (**C**) and 2:1:1 (**D**) wt. ratio obtained using spray drying.

In spray drying, the liquid is dispersed in a form of droplets and dried with a hot air. This leads to a formation of particles of consistent size distribution, usually of spherical or ruptured spheres shape of diameter below 10 micrometers. The data presented in Table 1 indicates that spray-dried binary systems exhibited particles of greater size that those obtained via evaporation technique. However, the span values (calculated using Equation (1)) are a bit smaller in case of spray-dried systems, which indicates that the distributions are narrower. The tails of the distribution suggest that the particles aggregated during the process, probably due to the fact of low melting temperature of poloxamer. This may also result from the fact that some amount of drying samples adhered to the inner wall of spray dryer, which additionally leads to the decrease in the process yield. The Dx(90) value of particles of PLX 188-based (SD) solid dispersions are bigger than systems containing PLX407. Interestingly, BCL-PLX407 2:1 (SD) system exhibited the smallest particles among all investigated systems, as seen in Figure 2B and the SEM image (Figure 3). The size distributions of PLX407-based solid dispersions were narrower, with well resolved maxima as compared to those obtained for systems containing PLX188. Moreover, the maximum of particle size distribution of the system containing twice as much bicalutamide as PLX407 in binary solid dispersions was shifted towards bigger particles (Dx(10) = 126.0 µm and Dx(90) = 380.0 µm). All of examined systems exhibited a tailed distribution towards lower values of particle size.

(1) $$Span=\frac{D_{x}\left(90\right)-D_{x}\left(10\right)}{D_{x}\left(50\right)}$$

Interestingly, the addition of PVP to BCL-PLX systems led to the formation of fine powders with the particle size distribution maxima located between 50 and 120 µm. However, the span reaches greater values than for binary solid dispersions, which may be a consequence of the distributions tailed towards smaller particles. Obtained ternary systems were also characterized with better flowability than platelet-like particles of binary solid dispersions. Particle size distributions of all the systems were more unified; moreover, the formation of a fraction of particles of size below 1 µm was also noticeable (Figure 1C).

An SEM analysis indicates that the crystals of neat bicalutamide adopt hexagonal shape and particles of smooth surface exhibiting ca. 160 µm in length [57]. The evaporation process led to the noticeable changes in the surface and morphology of obtained binary solid dispersions. During the rotation of the flask, the surface area of solvent increases. This leads to an enhancement of evaporation rate and fast recrystallization of dissolved bicalutamide. Thus, the formation of sharp-edged aggregates not exceeding 100 µm in case of BCL-PLX407 1:1 (E) and 200 µm for the other systems was observed (Figure 2). The systems comprised particles of wide size distribution, as seen in the SEM images as well as plots obtained using laser diffraction technique (Figure 1A).

A spray drying process usually leads to the formation of spherical particles with a consistent size distribution. The SEM micrographs of ternary solid dispersions show that obtained particles formed spheres of diameter not exceeding several microns, however they tended to agglomerate (Figure 3). In combination with the recrystallization that also occurred it led to the particle size distribution determined by means of laser diffraction measurements being much greater as the Dx(90) values varied between 227 µm and 273 µm.

#### 3.1.2. X-Ray Powder Diffractometry (XRPD)

**Figure 4:**
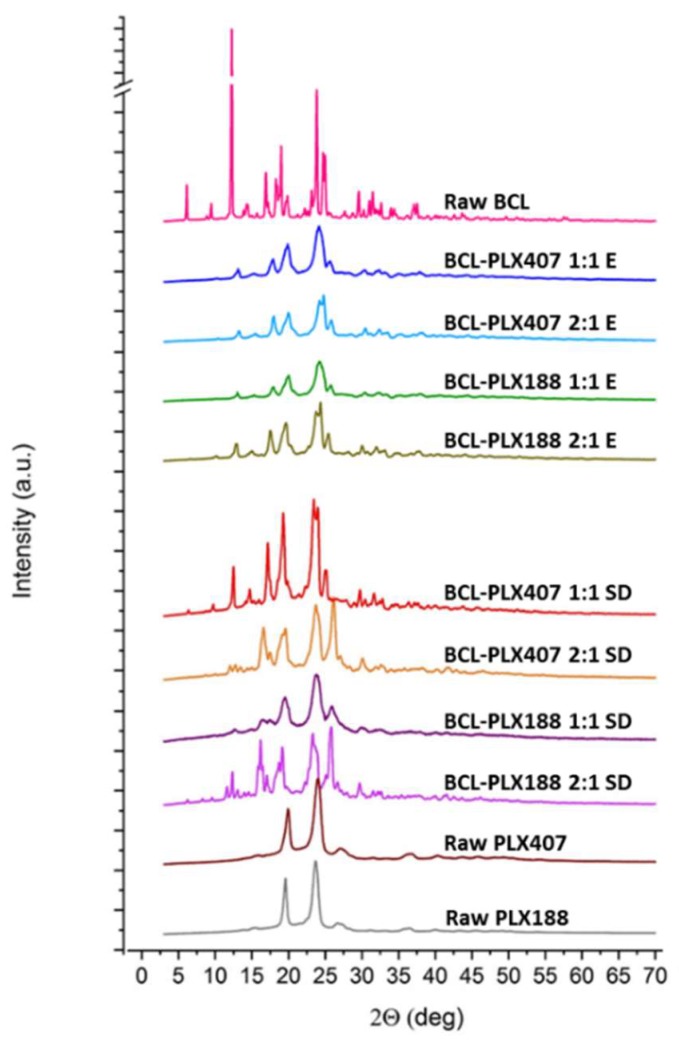
X-ray diffraction patterns of binary systems containing bicalutamide and either PLX 188 or PLX 407 (1:1 and 2:1 wt. ratio) obtained using evaporation technique (E) and spray drying (SD).

The XRPD studies were performed to characterize the molecular structure of binary systems. Obtained results indicate that both of applied processes led to the changes in molecular structure of the systems (Figure 4). The diffraction pattern of raw bicalutamide indicated by numerous distinctive Braggs peaks (2θ = 12.18°, 16.88°, 18.92°, 23.82°, 24.66°, and 24.94°) confirms that the drug exhibited highly-ordered arrangement on molecular level. The data confirm that bicalutamide existed as a form I polymorph (according to the 2014 Cambridge Crystallographic Data Centre (CCDC)). The decrease in crystallinity of the drug after co-processing with poloxamers is manifested by the reduction of the relative intensities of peaks. This suggests that the crystal lattice was partially destructed during processing. Moreover, the crystalline diffraction peaks are superimposed on the slightly noticeable amorphous halos. This indicates that the sample is amorphous to a very small extent. No transition to metastable polymorph was observed as no shifts in diffraction peaks appeared. This confirms that used poloxamers did not stabilize the disordered system at low concentration, in agreement with a previously published paper [59].

**Figure 5:**
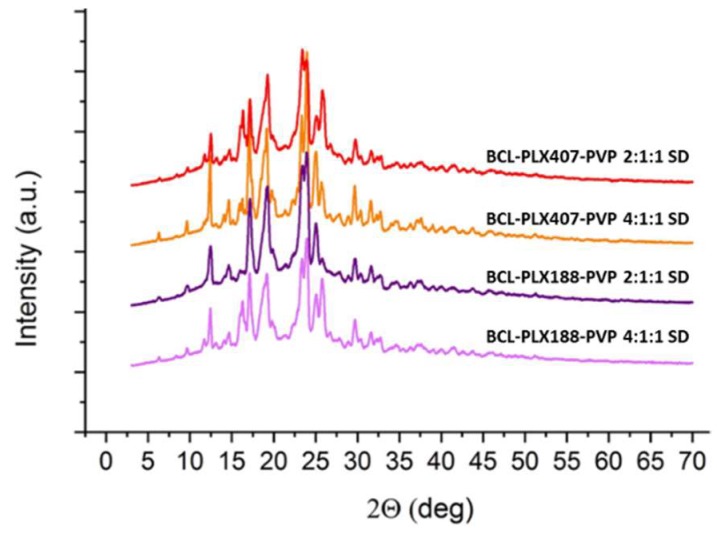
X-ray diffraction patterns of ternary systems containing bicalutamide, PVP, and either PLX 188 or PLX 407 obtained by spray drying.

Interestingly, the diffraction patterns were more structured in spray-dried systems than evaporated ones. Moreover, the obtained ternary solid dispersions exhibited one more important feature, the transition of BCL from form I into form II polymorph [60]. This is clearly marked in the diffractograms presented in Figure 5 and manifested by the additional intense peak between 25.08° and 25.86° 2θ, which does not appear in the diffractogram of raw BCL. No such solid–solid transition of bicalutamide–poloxamer solid dispersions has been described so far. The diffraction patterns also suggest that ternary systems contain a fraction of amorphous phase as the diffractograms are superimposed on the amorphous halo further assigned to partial amorphization of poloxamers (see Section 3.1.4).

#### 3.1.3. Vibrational Spectroscopy

**Figure 6:**
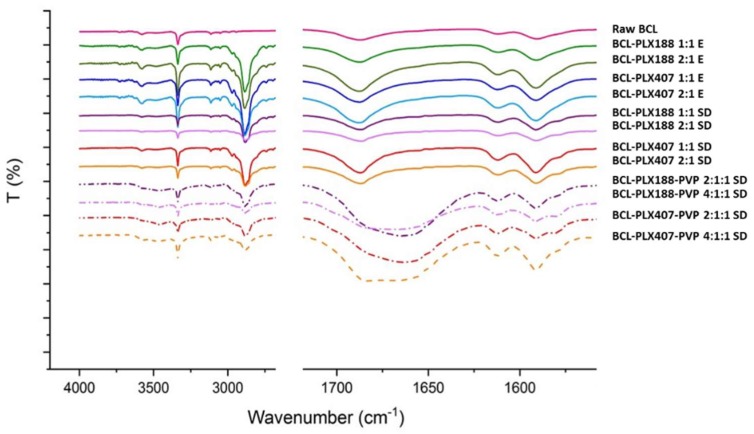
FTIR spectra of raw bicalutamide and binary and ternary solid dispersions.

FTIR spectroscopy has been applied to determine the molecular structure and possible interactions between BCL and the carriers in solid dispersions. The intensity, shape and position of peaks (the presence of shifts) were evaluated with an emphasis placed on the vibrations within the carbonyl and amine functional groups (Figure 6). Well-resolved bands at 3335 cm^−1^ correspond to N–H stretching vibrations, and the broad band with a maximum at 1687 cm^−1^ originates in C=O stretching vibrations. The spectra of binary solid dispersions do not differ significantly from those of pure drug, suggesting that BCL does not interact with any of used poloxamers or that the strength of the interactions is negligibly small. The new band that appears in the range of 2860–3000 cm^−1^ corresponds to the stretching vibrations of aliphatic C–H group in poloxamers.

Noticeable differences were observed for ternary solid dispersions as the band corresponding to carbonyl group vibration is broadened and the maximum red-shifted. This indicates the existence of strong intermolecular interactions between BCL and PVP as we previously showed [57]. Moreover, the O–H band is fuzzy, which confirms partial amorphization of the system.

#### 3.1.4. Thermal Properties of Solid Dispersions

**Figure 7:**
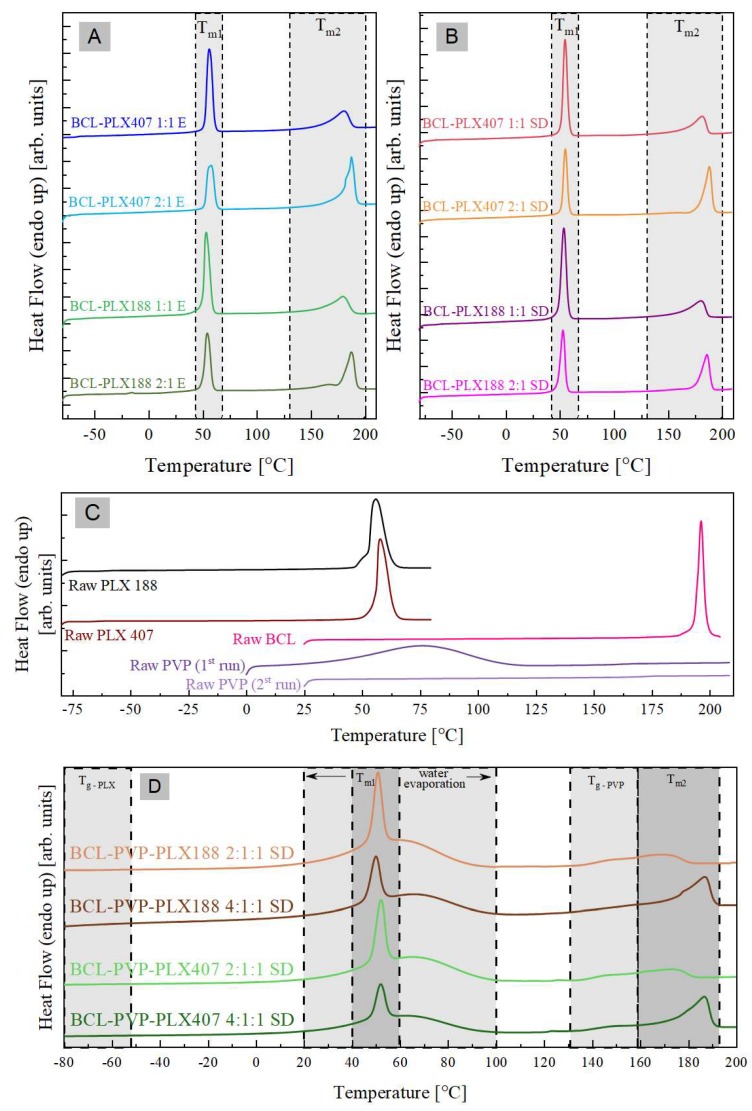
The DSC thermograms of: binary systems containing bicalutamide and either PLX188 or PLX407 obtained using the evaporation method (**A**) and spray drying (**B**), raw bicalutamide and polymers (**C**), and ternary spray-dried systems containing BCL, poloxamer, and PVP (**D**).

The thermal properties of raw systems (i.e., BCL, PLX188, PLX407, and PVP), the binary formulations containing BCL and either PLX407 or PLX188 polymer (that were obtained by two different methods—evaporation (E) and spray drying (SD)), and the ternary, spray-dried formulations of BCL, PLX, and PVP have been examined by means of the differential scanning calorimetry (DSC) technique. The DSC curves obtained during heating with a rate equal to 10 °C/min are presented in Figure 7.

As can be seen in the panel (C) of Figure 7, the DSC trace of raw BCL reveals a single sharp peak with an onset at 194 °C. This endothermal process corresponds to the melting of the investigated antiandrogen and is in a perfect agreement with the literature data [61]. Both DSC curves of PLX188 as well as PLX 407 exhibit two thermal events. The first (barely visible on the DSC thermograms presented in Figure 7C) is step-like transition occurring in the vicinity of −60 °C associated with the glass transition of the amorphous part of PLXs (poly(propylene oxide), PEO blocks). The second, located at around 50 °C, is a sharp endothermal peak originating from the melting of the crystalline part of the polymers (poly(propylene oxide), PPO block). Two thermal events have been also observed in the DSC trace of the neat PVP polymer, when measured as received. The first, very broad, thermal event that is located in the range of 20–100 °C is associated with water evaporation (note the absence of this process, when the sample is re-heated). The second step-like transition (barely visible in Figure 7C) occurring in the vicinity of 172 °C is associated with the polymer glass transition.

**Figure 8:**
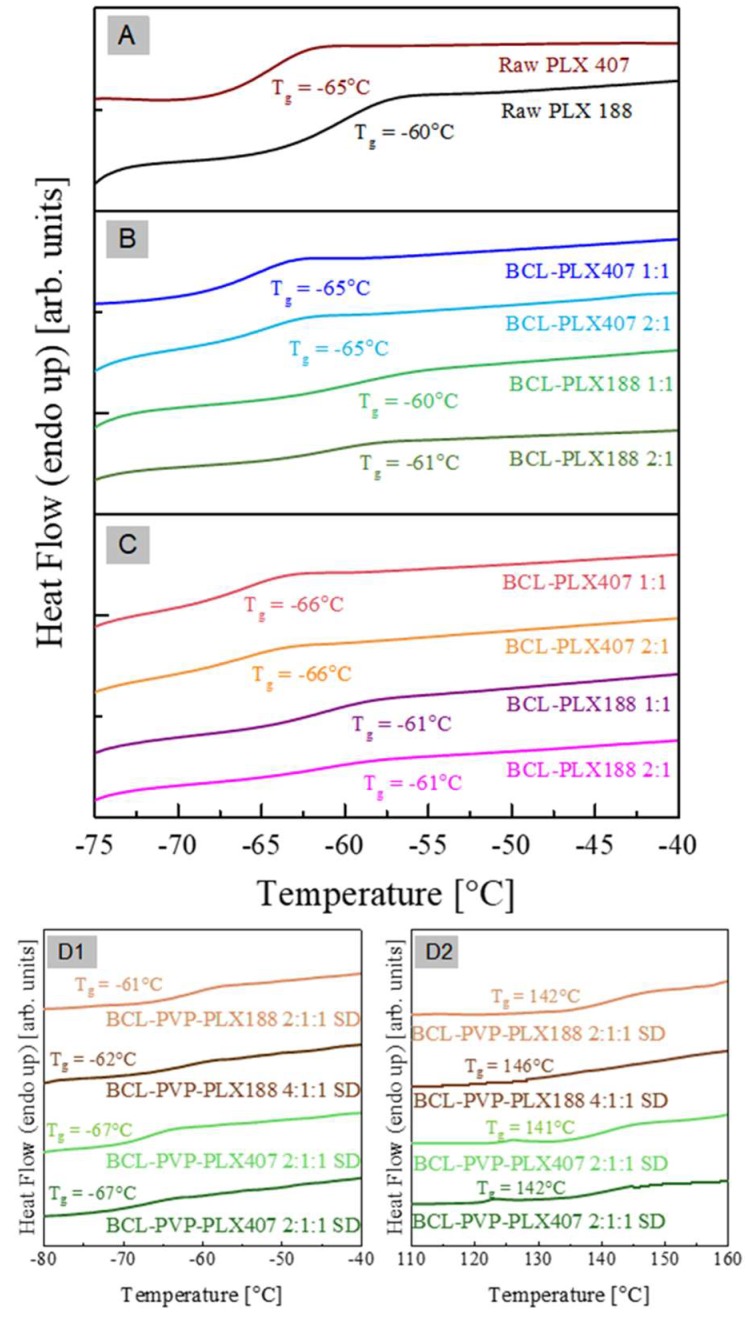
The zoomed fragment of DSC thermograms presented in the Figure 7 of raw bicalutamide and poloxamers (**A**), binary systems containing bicalutamide and either PLX188 or PLX407 obtained using evaporation method (**B**) and spray drying (**C**), and ternary, spray-dried, systems containing BCL, poloxamer, and PVP (**D1**,**D2**).

In the panels (A) and (B) of Figure 7, the DSC traces of binary drug-polymer compositions prepared by evaporation (panel A), and spray drying (panel B) are shown. As can be seen all investigated formulations reveals three thermal events—T_g_, T_m1_, and T_m2_—in the temperature range from −80 °C to 210 °C. Because the glass transition event (T_g_) is almost invisible in the scale of Figure 7, the data from the temperature region: −75 °C to −40 °C are presented in a separate figure (see Figure 8). In Table 2 the values of all investigated thermal events of all examined systems have been collected..

Since (1) the glass transitions of BCL-PLX 188 systems are located at the same temperature as the T_g_ of raw PLX 188, and (2) the glass transitions of the BCL-PLX 407 systems are located at the same temperature as the T_g_ of raw PLX 407, one can conclude that the glass transition event registered in binary formulations originates from the amorphous fraction of the PLXs (poly(ethylene oxide) PEO blocks). Comparing the values of the onsets of the thermal events which have been marketed in Figure 7A,B as T_m1_, one can identify them as the melting of the crystalline part of the polymer which exists in the solid dispersions. The third thermal event that has been registered during the DSC measurements of the BCL-PLX systems is located in the temperature range from 130 °C to 200 °C. This endothermal peak corresponds to the melting of the BCL contained in the solid dispersions. Therefore, one can observed that its enthalpy (ΔH_m2_) decreases with decreasing amounts of the BCL in the system. As can be seen, the onset of T_m2_ shifts towards lower temperatures with increasing amounts of PLX in the formulation. This might be connected with the dissolution of the drug in a liquid polymer.

In the panel (D) of Figure 7, the DSC traces of ternary drug–polymer–polymer compositions (prepared by spray drying) are shown. As can be seen, the investigated formulations reveal five thermal events which are marked in Figure 7D as T_g_-PLX, T_g_-PVP, T_m1_, T_m2_, and water evaporation. Since both T_g_-PLX and T_g_-PVP are almost invisible in the scale of Figure 7, the data from the temperature regions −80 °C to −40 °C and 110°C to 160°C are presented in the separate figures (see Figure 7 D1 and D2). From the comparison of the DSC traces of ternary systems to either raw and binary systems one can conclude that: (1) T_g_-PLX originates from the amorphous fraction of the PLXs (PEO blocks); (2) T_g_-PVP is associated with the glass transition temperature of PVP polymer; (3) T_m1_ reflects the melting of the crystalline part of the PLX polymer which exists in the system; and (4) T_m2_ corresponds to the melting of the BCL. Note that with increasing amount of API in the system, ΔH_m1_ and ΔH_m2_ are changing.

#### 3.1.5. Wettability of Solid Dispersions

Powder wettability is an important issue in pharmaceutical sciences as the solid–liquid interfacial interactions can affect drug dissolution, solubilization, and disintegration [62]. Given the heterogeneity of the surface properties resulting from a specific surface chemistry, variations between polymorphic and amorphous forms have been reported thus far [63]. They affect the level of supersaturation of molecularly-disordered systems and physical stability; thus, the assessment of wetting properties plays a significant role in the systems containing fine particles.

**Figure 9:**
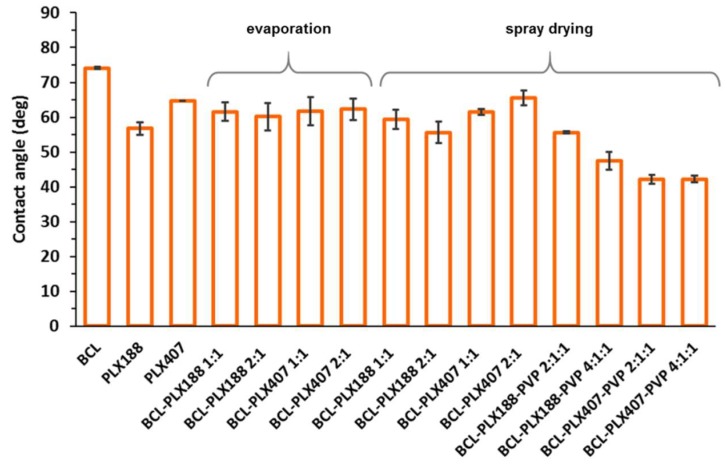
The values of contact angles of raw bicalutamide and poloxamers, binary and ternary systems containing bicalutamide, poloxamers, and PVP obtained using either evaporation method or spray drying.

The wetting behavior of raw compounds as well as binary and ternary solid dispersions were assessed by contact angle measurements using the sessile drop technique. The difference between the two used poloxamers is clearly visible (Figure 9). The values of measured contact angle were equal to 56.8 ± 1.8° and 64.7 ± 0.02° for PLX188 and PLX407, respectively. The difference may result from the differences in molecular composition of both polymers, i.e., higher amount of hydrophobic poly(propylene oxide) units and greater molar mass of PLX407 [49]. Interestingly, no significant effects of either the type of applied poloxamer or the process on the wettability of binary solid dispersions were observed. All the systems exhibited improved wettability expressed by the decreased contact angle in comparison with raw BCL (θ = 74.1 ± 0.3°) with slightly higher values determined for systems containing PLX407. Interestingly, the addition of PVP to ternary solid dispersions obtained by spray drying led to well pronounced increase in wetting behavior of the systems. While the values of contact angle for binary systems ranged between 60°–65°, for the systems comprising polyvinylpirrolidone they reached ca. 42°–45°. Moreover, the effect of molecular structure of poloxamers was less significant as lower values of the contact angle were obtained for PLX407-based systems. This is of particular importance as the improved wettability and surface activity of poloxamers can strongly affect the improvement in bicalutamide dissolution.

### 3.2. Characterization of Solid Dispersions in Solution

#### 3.2.1. Self-Assembly of Poloxamers in Solid Dispersions

The assembly phenomenon of amphiphilic polymers has been intensively studied in recent years [64,65,66]. Their aggregation leads to the formation of hydrophobic domains that are able to solubilize sparingly water-soluble molecules. This reduces the agglomeration of drug molecules and increases the dissolution of API.

Poloxamers are low-meltable triblock copolymers consisting of hydrophobic chain of poly(propylene oxide) bound with two hydrophilic chains of poly(ethylene oxide) able to solubilize hydrophobic molecules [67]. While PLX188 contains ca. 15% of PPO, PLX407 is composed of ca. 35% of PPO, which may affect the assembly of copolymer in polar media [68].

**Figure 10:**
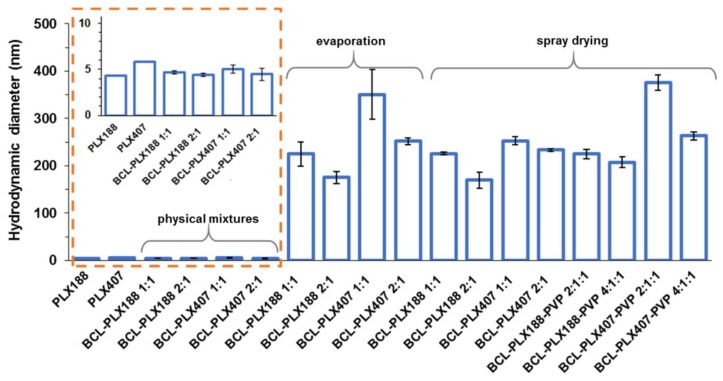
Hydrodynamic diameters of aggregates formed in aqueous solutions of poloxamers, physical mixtures, and binary systems obtained using either the evaporation method or spray drying. Insert: zoomed data corresponding to raw PLXs and physical mixtures.

The size of molecular assemblies of both used poloxamers did not exceed 6 nm, however the diameter of PLX407-based particles is ca. 30% greater than those formed by PLX188, which may result from the higher content of PPO units. Physical mixtures also assembled in particles of diameter below 6 nm; however, the mixtures containing equal amounts of BCL and PLX formed particles of ca. 10–12% greater diameter than those containing the excess of the drug (Figure 10).

The mean hydrodynamic diameters of all solid dispersions based on PLX188 are smaller than those containing PLX407, regardless of the method of preparation and the number of system constituents. Moreover, solid dispersions containing 50% of the carrier exhibited aggregates of greater diameter than those with the excess of bicalutamide, similarly to physical mixtures. However, the differences reached up to 39% for the BCL-PLX407 1:1 (E) system. No significant variations occurred between the PLX188-based binary systems of corresponding compositions obtained by the two methods. The systems containing the excess of the drug exhibited particles of ca. 170 nm in diameter, while the size of aggregates formed by 1:1 systems was equal to 225 nm. The great variation in particle size was noticed in BCL-PLX407 systems, especially the evaporated one containing an equal amount of the drug and the carrier. The diameter of these particles reached 350 nm in diameter, while the aggregates of spray-dried solid dispersion did not exceed 250 nm in diameter (Figure 10). Similar behavior was observed for BCL-PLX407-PVP 2:1:1 (SD) which exhibited particles much greater that the other ternary systems. The addition of PVP to solid dispersions did not affect the self-assembly behavior. The differences in hydrodynamic diameters values follows the same trend as for binary systems, with slightly greater values for solid dispersions containing equal amount of BCL and the carriers (PLX and PVP).

**Figure 11:**
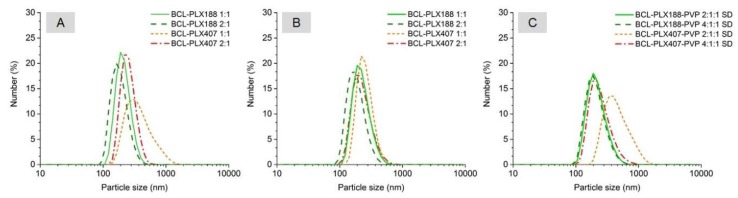
Number-weighted particle size distribution of aqueous solutions of evaporated (**A**), spray-dried binary (**B**), and ternary (**C**) solid dispersions.

The DLS measurements confirmed monomodal and rather narrow distribution of particle size (Figure 11) with maxima slightly shifted towards greater values for PLX407-based systems, regardless of the method of solid dispersion preparation. The long tail of distribution of the BCL-PLX407 1:1 (E) binary system assigned to the formation of several aggregated structures that disrupted the measurement explains the variation in particle size in comparison with the other systems. The size of the formed aggregates was determined to be almost 40% greater than for BCL-PLX407 2:1 (E) system, similarly to BCL-PLX407-PVP 2:1:1 (SD) compared with BCL-PLX407-PVP 4:1:1 (SD) solid dispersions. This confirms that the effect of the applied method of solid dispersion preparation is negligible when considering that the solution and the composition are the most important factors.

#### 3.2.2. Solubilization of Molecular Probes

Fluorescence spectroscopy has been applied to the determination of micropolarity, microviscosity and solubilization ability. Two molecular probes were used due to the act that their fluorescent properties vary depending on physical parameters of nanoassemblies.

**Figure 12:**
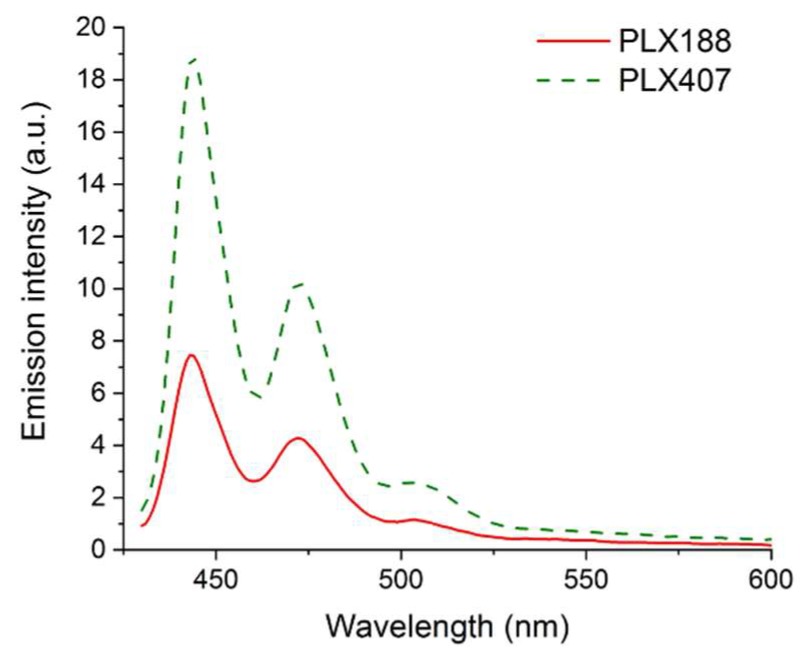
Emission spectra of perylene solubilized by either Poloxamer^®^188 or Poloxamer^®^407 solution (λ_ex_ = 405 nm).

Perylene exhibits unique properties, i.e., low solubility in water (c = 1.6 × 10^−9^ M) and lack of fluorescence in polar environment [69]. The presence of characteristic emission bands indicates that the probe experienced non-polar environment and confirms that the probe is solubilized within hydrophobic packets formed by self-assembled poloxamer molecules (Figure 12).

The emission spectrum of pyrene yields in the information about the polarity sensed by the probe in the solubilization site. The intensity of the vibronic fine structure of the monomeric form of pyrene depends on the polarity of the microenvironment [70]. In polar media there is an increase in the intensity of the 0-0 band (peak I), whereas band III is affected only slightly [71]. The ratio of the emission intensities I_III_ (at 386 nm) and I_I_ (at 374 nm) was used to study environmental changes experienced by the probe. The values presented in Table 3 indicate that the probe experienced less polar environment while solubilized within any of examined system in comparison to water. However, the increase in the I_III_ to I_I_ ratio is rather low in solid dispersions (especially the spray-dried ones), which suggests that the systems formed loosely packed nanoasseblies easily penetrable by water molecules.

#### 3.2.3. Dissolution Study

The methods aimed at the enhancement of the dissolution of bicalutamide have been already considered in several papers. Solvent evaporation under reduced pressure was applied to obtain solid dispersions containing bicalutamide and PVP in 1:3, 1:4, and 1:5 drug-to-polymer ratios, respectively [59,72]. The formation of binary systems led to the amorphization of BCL; however, a great excess of PVP was required. The authors concluded that such a high proportion of the carrier may lead to the increase in bulkiness and tablet weight during the development of a formulation. Solid dispersions with poloxamer were obtained by melting [73] and supercritical carbon dioxide method [74]. The samples containing 83.8% of the carrier were found to be amorphous, however gelling properties of PLX retarded the dissolution of the drug from the systems containing high concentration of the polymer. The increase in the amount of poloxamer in solid dispersions was concluded not to offer any advantage for the dissolution improvement.

**Figure 13:**
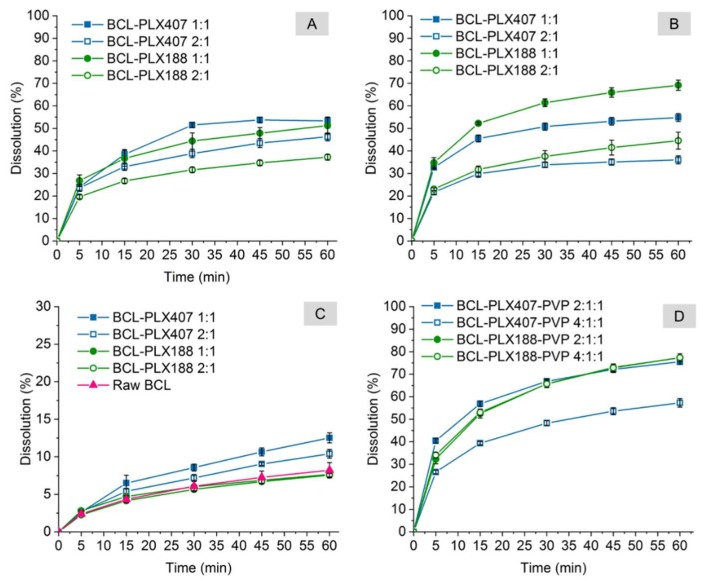
Dissolution of binary and ternary systems containing bicalutamide, poloxamers and PVP (in case of ternary systems) obtained using the evaporation technique (**A**), spray drying (**B**,**D**), and physical mixing (**C**).

Due to the aforementioned problems caused by the excess of poloxamer, we prepared binary and ternary systems containing either equal amounts of bicalutamide and polymer or twice as much BCL as the carriers. The dissolution profiles presented in Figure 13 showed a significant improvement (from 4- to 8-fold) of the drug dissolution in comparison with raw BCL and BCL-PLX physical mixtures. Only 8.2% of crystalline bicalutamide dissolved after 1 h of the dissolution test. Moreover, the formation of the systems in which BCL was physically mixed with the readily soluble carrier affected the dissolution of the drug only slightly as less than 12.6% of bicalutamide dissolved from physical mixture containing PLX407 and ca. 8% from PLX188-based systems (Figure 13C).

The dissolution profiles of solid dispersions were found to be independent on the applied processes. The amount of bicalutamide dissolved from binary systems processed in 2:1 wt. ratio varied between 36.0% for BCL-PLX407 (SD) and 37.2% for BCL-PLX188 (E) to 44.6% for BCL-PLX188 (SD) and 46.3% for BCL-PLX188 (E). Solid dispersions containing equal amounts of the drug and the carrier exhibited better dissolution than those containing the excess of the drug, as 51.3% of bicalutamide dissolved from BCL-PLX188 (E), 53.3%% from BCL-PLX407 (E), and 54.8% from BCL-PLX407 (SD). The variation was observed only for BCL-PLX188 1:1 (SD) solid dispersion as 69.6% of the drug dissolved after 1 h. Interestingly, dissolution curves obtained for spray-dried systems with both binary and ternary solid dispersions (Figure 13B,D) showed an opposite tendency in comparison to evaporated systems (Figure 13A), as in evaporated systems more bicalutamide dissolved from PLX407-based solid dispersions, while after spray drying, systems containing PLX188 exhibited better dissolution. Importantly, the addition of PVP seems to positively affect BCL dissolution, as 77% of BCL dissolved from both systems containing PLX188, while 75.6% and 57.3% dissolved from the 2:1:1 and 4:1:1 PLX407-based systems, respectively.

## 4. Conclusions

The obtained results indicate that co-processing of BCL with PLXs leads to an improvement of bicalutamide dissolution from 4- to 8-times in comparison with the pure drug. That effect was assigned to the formation of nanoaggregates. Surface activity of poloxamers leads to the formation of hydrophobic packets in which bicalutamide was solubilized. Importantly, physical mixtures did not form aggregates with bicalutamide and thus no significant enhancement in drug dissolution was observed. While no variations in dissolution between systems obtained by either spray drying or evaporation processes were noted, some differences in physicochemical characteristics appeared. The most important observation is that the drug partially lost its highly-ordered molecular structure after preparation of solid dispersions. The changes in diffractograms were more pronounced in evaporated systems. The decrease in crystallinity was expressed by the decrease in relative intensity and lack of several peaks. Moreover, the addition of PVP and formation of ternary solid dispersions by spray drying led to the transition of polymorph I into polymorphic form II of bicalutamide. This confirms that the interplay between the process parameters and properties of both drug and carrier is important to obtain solid dispersion of desired characteristics without a great excess of the auxiliary compounds.

The type of polymer was found to affect the size of nanoaggregates formed by solid dispersions in an aqueous medium. The self-assembly of systems containing PLX188 led to the formation of smaller particles, regardless the applied technique of solid dispersion preparation. This may be a result of the composition of the macromolecule, as it contains ca. 15% PPO hydrophobic mers, while PLX407 contains ca. 35%.

Thermal analysis confirmed that poloxamers were partially amorphous in solid dispersions, which indicates that the drug antplasticizes the T_g_ of the polymer. This would be connected with the dissolution of the drug in a liquid polymer.

## Figures and Tables

**Table 1:** Particle size of solid dispersions obtained using the laser diffraction method. PLX188: Poloxamer^®^188, PLX407: Poloxamer^®^407.

Method	Carrier	BCL:polymers wt. Ratio	Dx(50) ± SD (µm)	Span
Evaporation	PLX188	1:1	247.0 ± 55.9	4.698
		2:1	227.0 ± 30.7	2.719
	PLX407	1:1	203.0 ± 23.1	2.971
		2:1	159.0 ± 53.1	4.438
Spray-drying	PLX188	1:1	196.0 ± 27.2	4.405
		2:1	355.0 ± 64.7	2.876
	PLX407	1:1	445.0 ± 28.7	1.771
		2:1	154.0 ± 13.7	2.167
	PLX188-PVP	2:1:1	78.0 ± 0.8	3.339
		4:1:1	55.6 ± 1.5	3.907
	PLX407-PVP	2:1:1	48.6 ± 2.2	5.153
		4:1:1	54.2 ± 2.1	4.098

**Table 2:** Comparison of the T_g_, T_m1_, and T_m2_ values of raw BCL, poloxamers, PVP, binary systems containing bicalutamide and either PLX188 or PLX407 (obtained using evaporation method (E) and spray drying (SD)), and ternary systems containing bicalutamide, PLX, and PVP.

System	T_g-PLX_ (°C) (midpoint)	T_g-PVP_ (°C) (midpoint)	T_m1_ (°C) (onset)	ΔH_m1_ (J/g)	T_m2_ (°C) (onset)	ΔH_m2_ (J/g)
Raw BCL	-	-	-	-	194	110.8
Raw PVP	-	172	-	-	-	-
Raw PLX 188	−60	-	53	134.9	-	-
Raw PLX 407	−65	-	56	117.3	-	-
BCL-PLX 188 1:1 E	−60	-	50	65.7	160	39.6
BCL-PLX 188 2:1 E	−61	-	49	45.7	176	42.4
BCL-PLX 188 1:1 SD	−61	-	49	66.6	160	39.2
BCL-PLX 188 2:1 SD	−61	-	48	42.3	176	42.6
BCL-PLX 407 1:1 E	−65	-	52	59.6	160	39.5
BCL-PLX 407 2:1 E	−65	-	52	39.2	182	52.4
BCL-PLX 407 1:1 SD	−66	-	52	58.9	165	39.6
BCL-PLX 407 2:1 SD	−66	-	52	38.9	181	49.2
BCL-PLX-PVP 188 2:1:1 SD	−61	142	47	188	135	27
BCL-PLX-PVP 188 4:1:1 SD	−62	146	45	120	134	50
BCL-PLX-PVP 407 2:1:1 SD	−67	141	47	140	135	26
BCL-PLX-PVP 407 4:1:1 SD	−67	142	47	95	137	48

**Table 3:** The I_III_ to I_I_ ratio calculated based on the fluorescence emission spectra of pyrene solubilized in aqueous solutions of either pure compounds or solid dispersions (λ_ex_ = 330 nm).

System	I_III_/I_I_	System	I_III_/I_I_
Water	0.351	BCL-PLX 188 1:1 (SD)	0.442
Raw PLX188	0.516	BCL-PLX 188 2:1 (SD)	0.430
Raw PLX407	0.503	BCL-PLX 407 1:1 (SD)	0.435
		BCL-PLX 407 2:1 (SD)	0.442
BCL-PLX 188 1:1 (E)	0.508	BCL-PLX 188-PVP 2:1:1 (SD)	0.556
BCL-PLX 188 2:1 (E)	0.523	BCL-PLX 188-PVP 4:1:1 (SD)	0.628
BCL-PLX 407 1:1 (E)	0.441	BCL-PLX 407-PVP 2:1:1 (SD)	0.661
BCL-PLX 407 2:1 (E)	0.512	BCL-PLX 407-PVP 4:1:1 (SD)	0.670
